# Electrolytic plasma processing-an innovative treatment for surface modification of 304 stainless steel

**DOI:** 10.1038/s41598-017-00204-w

**Published:** 2017-03-22

**Authors:** Wanyuan Gui, Junpin Lin, Guojian Hao, Yuhai Qu, Yongfeng Liang, Hui Zhang

**Affiliations:** 1 State Key Laboratory for Advanced Metals and Materials, University of Science and Technology Beijing, Beijing, 100083 China; 2 Qian Xuesen Laboratory of Space Technology, China Academy of Space Technology, Beijing, 100094 China

## Abstract

There is widespread attention to surface profile and modification of 304 stainless steel for research development and application. Here, a successful electrolytic plasma processing (EPP) technique has been developed for both surface pretreatment and coating deposition of 304 stainless steel. Representative images confirm that the number of the pits increases and the ravines gradually disappear on the steel pretreated by EPP with the increase of processing time and applied voltage. Moreover, there is an obvious enhancement in surface roughness of 304 stainless steel after EPP pretreatment. In the case of coating deposition, the further EPP modification conducted on the pretreated sample offers a simple and effective technique for the production of zinc coatings having the features of full coverage and homogeneous distribution. The results show that a zinc coating with a thickness of approximately 0.5 μm can be obtained on the 304 stainless steel by means of EPP for only 60 s.

## Introduction

The surface profile and properties of 304 stainless steel have been a great concern of research development and application in many fields, such as vehicle industry, desalination, and construction, etc, considering contaminant adhesion, corrosion issues, and performance optimization^1–5^. In particular, zinc coatings on the steel surface have been widely developed and used in order to provide corrosion protection and enlarge service life of the steel for industrial applications. However, conventionally galvanized methods, mainly including electroplating and hot dipping, have disadvantages of unfriend environment, or high energy consumption, etc refs 6–16. Moreover, there is a close correlation of the coating quality (e.g. adhesion and uniformity) to surface pretreatment associated with other processes such as acid pickling and ultrasonic cavitation etc. Thus, a new technique in a simple and competitive way has been greatly inspired to occur for surface modification of the steel.

Attempts have been made to use electrolytic plasma processing (EPP) as a relatively innovative means of surface treatment and coating for improving specific surface topographies and coating quality of metallic materials^17–23^. The formation of plasma on the surface of anodic or cathodic substrate, which depends on the polarity of applied voltage, gives the capability to carry out anodic or cathodic EPP for various surface engineering. For the anodic EPP, it has been usually associated with surface passivation and coating, leading to oxidizing, carburizing, and/or nitriding of the electrode surface^24–26^. While the cathodic EPP has been used for preparing metal nanoparticles or depositing metallic coating^27, 28^. Moreover, it has been realized that the cathodic EPP can both remove surface contaminants and boost filming kinetics, as it overcomes the issues of traditional methods. A good feasibility to form Zn or Al coating on the high Nb-TiAl alloy and steel via the cathodic EPP has been also confirmed, according to thermodynamic analysis and simulation in our previous work^20^. However, there is a lack of experimental evidences for surface modification and coating features on the metallic materials.

In this work, EPP is extended to pretreat the steel surfaces and deposit the zinc coatings on 304 stainless steel. The main aims here are thereby to verify that EPP can be effectively carried out for both surface pretreatment and coating deposit on 304 stainless steel, and to investigate the influence of EPP parameters (i.e. processing time and applied potential) on surface microstructure and features of the steel surface and zinc coating. The results obtained can provide practical guidance of a highly effective and efficient EPP way in surface modification of 304 stainless steel and other metallic materials for favorable application.

## Results

EPP has been successfully employed for both surface pretreatment and coating deposition of 304 stainless steel. The effect of EPP duration and applied voltage have been also studied on the surface microstructure and characteristics of the steel. It can be confirmed that there is an enhancement in the size and quantity of craters on the steel pretreated by EPP with the increase of processing time and applied voltage due to the increased number and intensity of plasma discharge. In the meanwhile, surface roughness of 304 stainless steel after surface pretreatment of EPP also increases, suggesting that the pretreated surface could provide favorable feasibility for the coating growth with good adhesion. Thus, a zinc coating with a full coverage and homogeneous distribution after further EPP treatment has been verified to form on the surface of the pretreated steel. More importantly, the surface modification of 304 stainless steel can be performed in a highly efficient and effective way by means of EPP.

## Discussion

### Surface morphology

**Figure 1:**
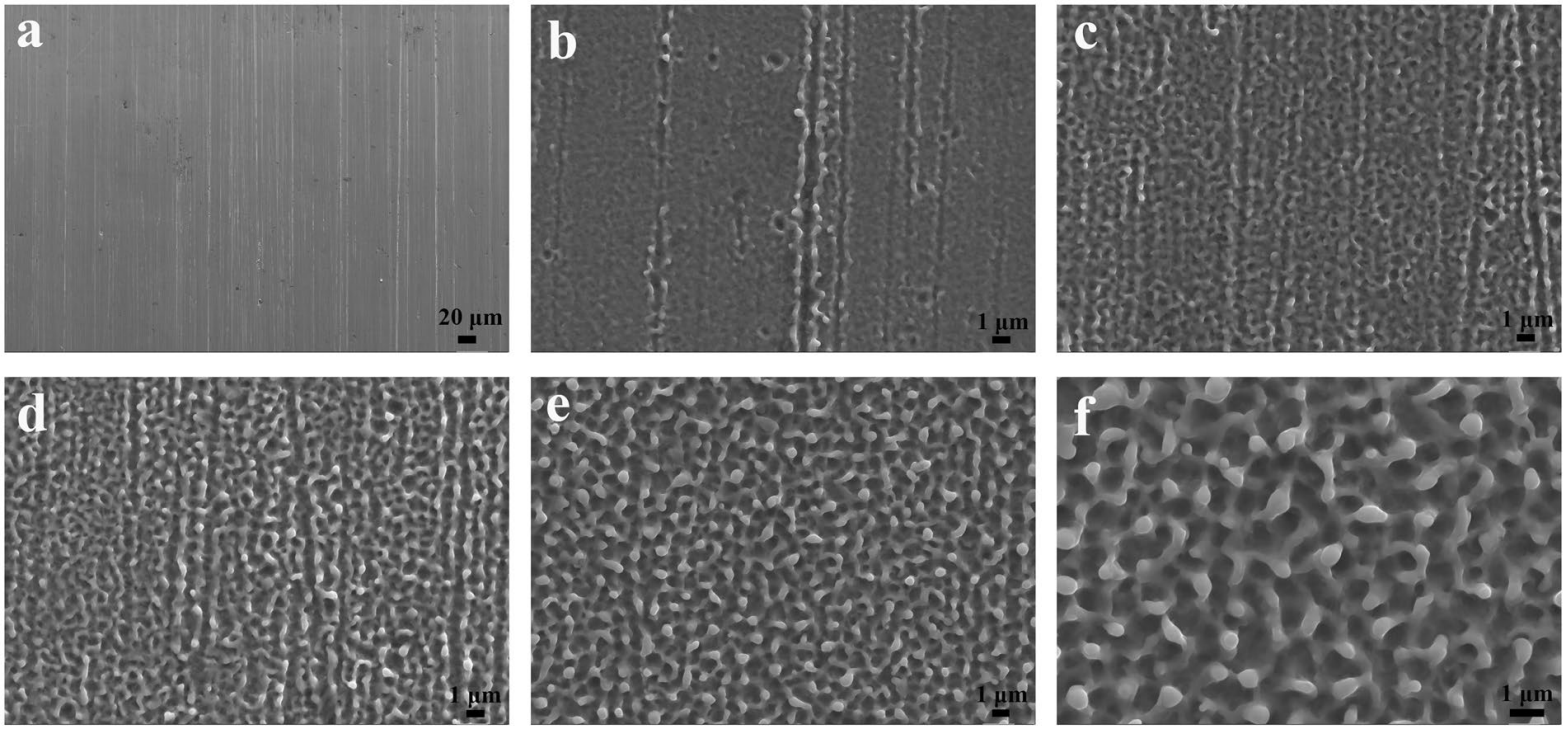
FESEM image of 304 stainless steel after EPP at an applied potential of 110 V for various intervals: (**a**) 0 s; (**b**) 20 s; (**c**) 40 s; (**d**) 60 s; (**e**) 120 s; and (**f**) 180 s.

**Figure 2:**
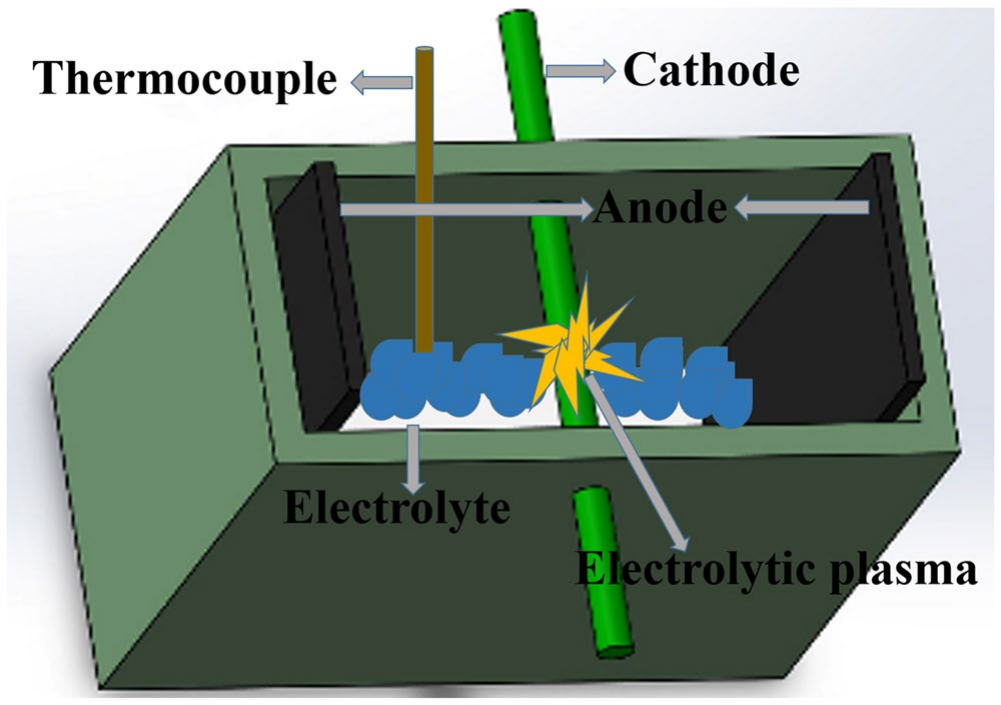
Schematic illustration of surface modification via EPP.

Differences are observed concerning the surface morphology of 304 stainless steel treated by EPP at an applied potential of 110 V with various processing time (0–180 s) as given in Fig. 1. The surface of the untreated 304 stainless steel before EPP displays clear ravine-like texture. When operating EPP at 110 V, a strong electric field can be formed across the electrode gap, leading to the evolution of mass hydrogens at the surface of the workpiece. The hydrogen plasma is thereby generated in a thin electrolyte layer just above the sample surface as shown in Fig. 2. After EPP treatment of 20 s, sharp edges on the steel surface tends to be smooth and surface craters occur. This can be attributed to local melting of the steel surface resulted from joule heat and hydrodynamic instability caused by solvent electrolysis. In the meantime, the generation and extinction of hydrogen bubbles created forceful pressure and shock wave at the surface, removing surface scale and contaminations. As reaction time extends, the surface becomes more rough and mossy, as well as the size and quantity of craters arise because of the increased number of plasma discharge channels releasing a large amount of energy on the surface^29–32^. When the EPP treatment increases 180 s, shown in Fig. 1(f) is the uniform distribution of globules and pits in a typically intersectional characteristics. The diameters of the globules and pits are about 0.3–0.5 μm and 0.5–0.8 um, respectively. The consequence above can be explained by the formation and effect mechanism of plasma in the reports for other metallic materials^33^.

**Figure 3:**
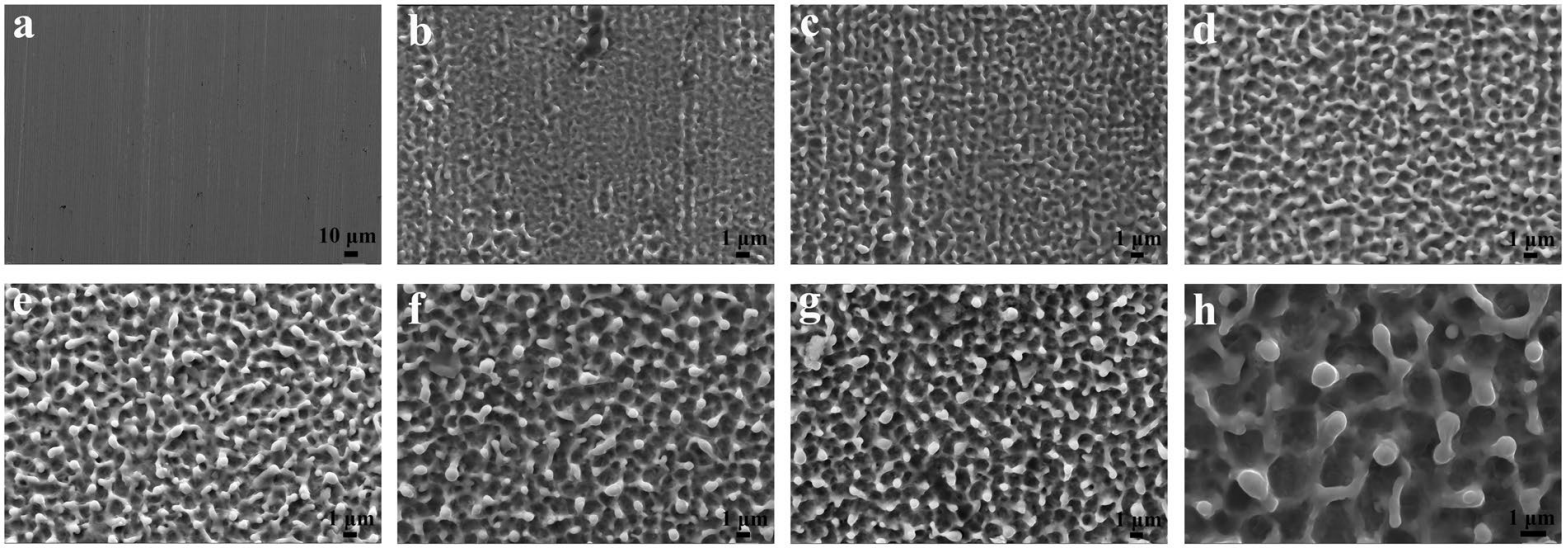
FESEM image of 304 stainless steel treated by EPP through using various applied potentials and intervals: (**a**) 120 V, 0 s; (b) 120 V, 20 s; (**c**) 120 V, 40 s; (**d**) 120 V, 60 s; (**e**) 120 V, 120 s; (**f**) 120 V, 180 s; (**g**) 110 V, 180 s; and (**h**) 130 V, 180 s.

Figure 3 displays the surface micrographs of the samples treated by EPP to determine the effect of applied potential and processing time on the steel surface. In the case of time extension, there is a similar trend to the results in Fig. 1, showing a gradual increase in size of microstructural crater and produced area of mossy surface. With an increase of the applied voltage from 110 V to 130 V with the same reaction time (180 s), as shown in Fig. 3(f–h), the diameters of the globules and pits increase more than doubles. These results can be attributed to the increase of discharge voltage and discharge energy, as a results, breakdown of electrolytic plasma bubbles constantly releases a large amount of energy and pressure on the sample surface^34, 35^.

### Three-dimensional morphology and surface roughness

**Figure 4:**
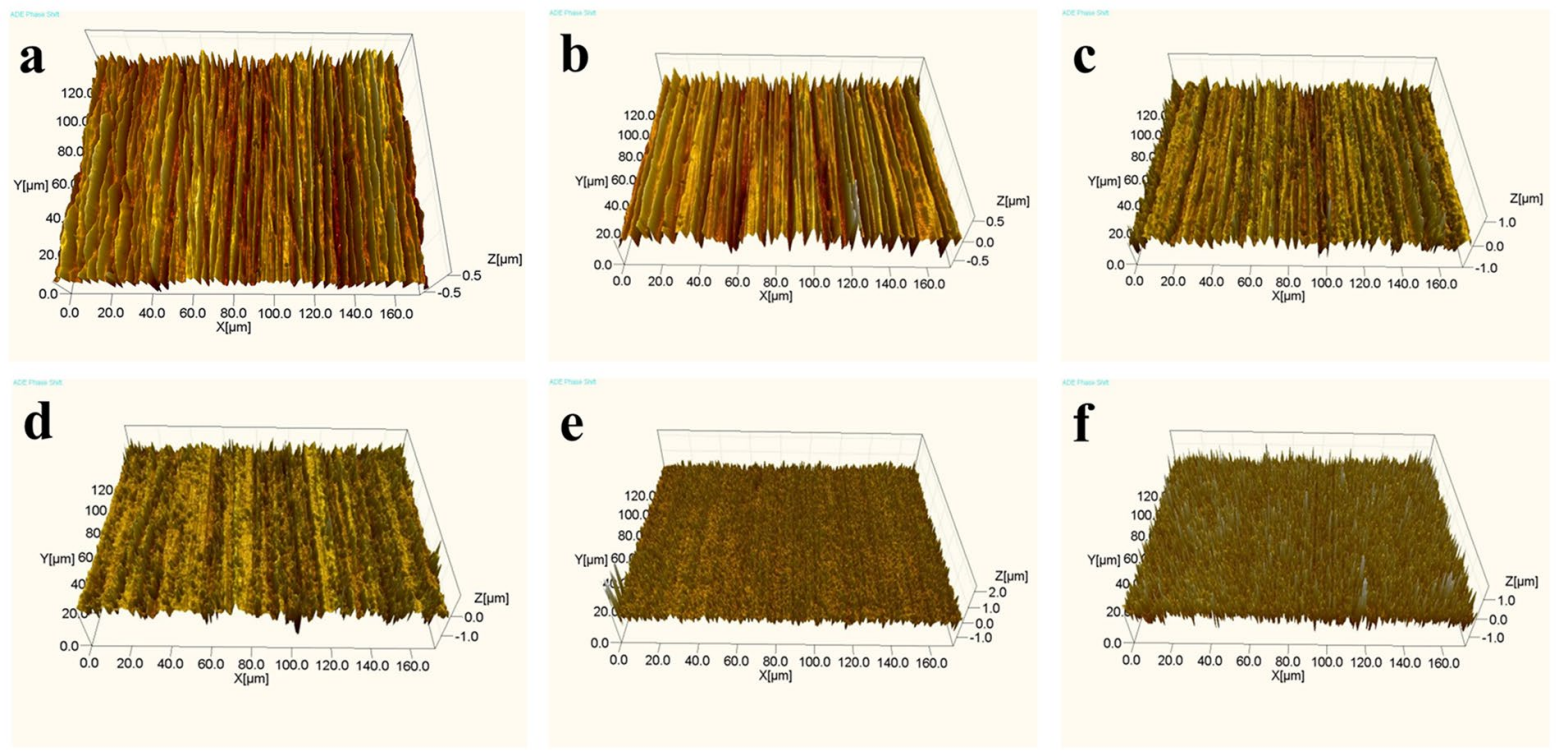
3D topography of 304 stainless steel before and after EPP with an input voltage of 120 V for different processing time: (**a**) 0 s; (**b**) 20 s; (**c**) 40 s; (**d**) 60 s; (**e**) 120 s; and (**f**) 180 s.

3D topography of 304 stainless steel treated by EPP with an input voltage of 120 V for various time are shown in Fig. 4. It can be verified that there is the same change trend of the 3D surface topography and surface morphology depicted above as a function of processing time. Before EPP treatment, there is clear ravine-like texture existing at the surface of the 304 stainless steel, as shown in Fig. 4(a). With the increase of EPP interval under an applied voltage of 120 V, height fluctuation becomes smaller in the microscopic texture and more pits form on the steel surface under the shock wave of hydrogen plasma. This suggests that the surface modified with the extension of EPP duration (e.g. 120 and 180 s) uniformly distributes abundant microholes, and the bulges and ravines practically disappear. It can be expected that the steel surface with uniform distribution of microholes could provide subsequent electroplating or EPP coating with good adhesion and uniformity.

**Figure 5:**
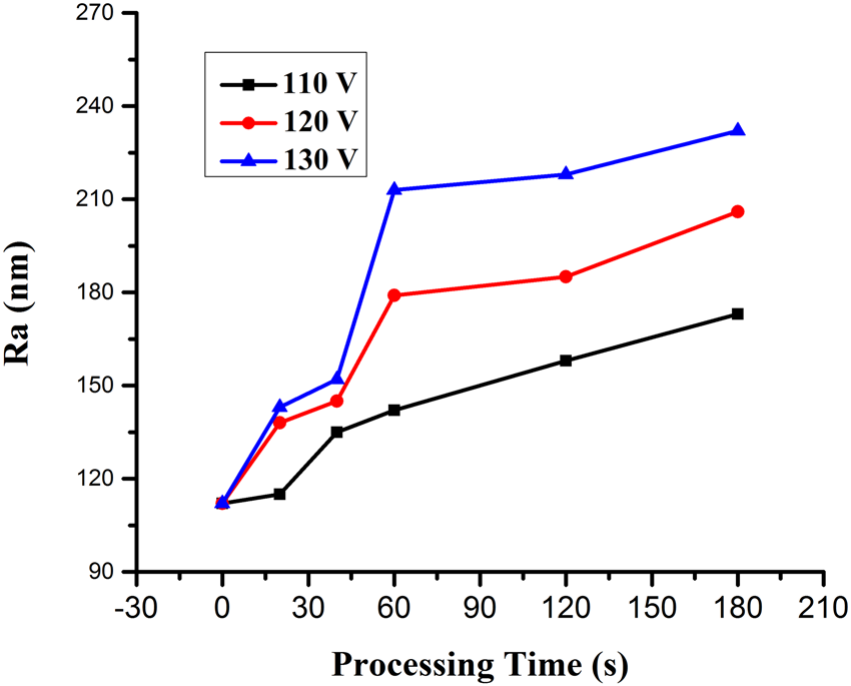
Surface roughness of 304 stainless steel as functions of EPP interval and applied voltage.

Surface roughness (*Ra*) of the steel modified by EPP is presented with the change of applied voltage and processing time are shown in Fig. 5. It can be confirmed that the *Ra* of 304 stainless steel increases with the enhancement of input voltage and reaction time. At the beginning, there is not enough energy to form amount of electrolytic plasma, as a results there is small change in *Ra* of the steel. As the reaction process continues, the breakdown of electrolytic plasma bubbles constantly releases a large amount of energy on the sample surface, while the *Ra* of 304 stainless steel moved up rapidly, which is consistent with the results of S K Sengupta^36^. In addition, solution volatilization caused by joule heat and solution hydrodynamic instability caused by solvent electrolysis of EPP lead to the considerable growth of partial melting areas of the steel surface and the formation of multiple discharging channels. All of these have a great effect on the increase of discharge hole diameter (i.e., roughness). Therefore, the maximum EPP time in the experiment, i.e. 180 s, makes the occurrence of the most craters on the steel surface with a greatest Ra value.

### Hardness testing

**Figure 6:**
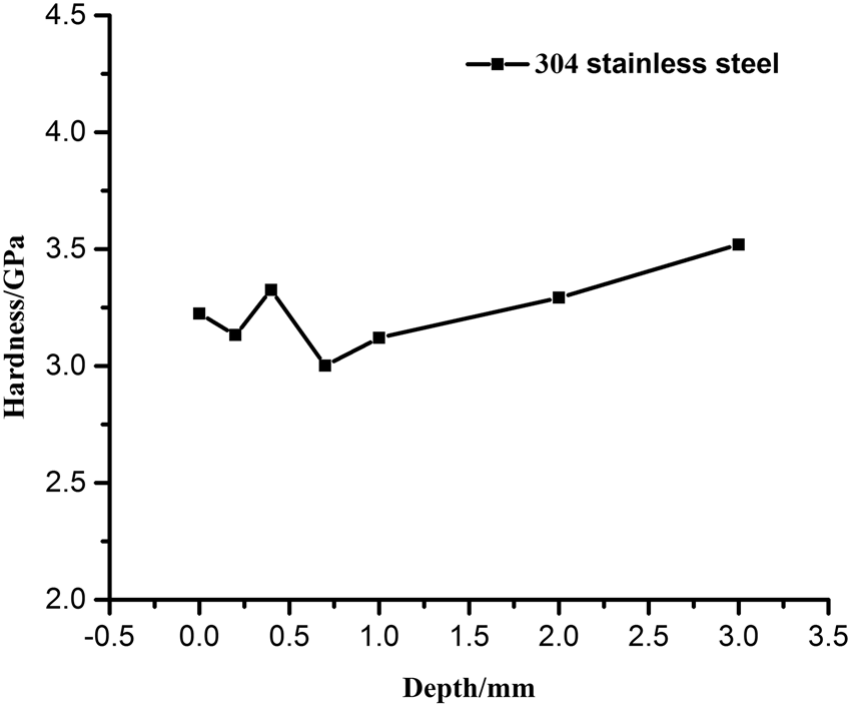
Changes in hardness along the surface to core radius of 304 stainless steel treated by EPP for 60 s with 120 V applied voltage.

The mechanical properties of alloy surface could be affected by electrolytic plasma reactions with high temperatures and strong shock waves^20, 30^. We employs nanoindentation testing to measure the hardness of 304 stainless steel treated by EPP for 60 s with 120 V applied voltage, as shown in Fig. 6, in order to quantitatively characterize the effect of the electrolytic plasma on the sample surface. The results demonstrated that EPP affects the surface mechanical properties mainly within the depth of 1 mm from the surface to core. The average hardness of the subsurface with the distance of 1 mm from the surface slightly decreases after EPP treatment, because the surface structures grow loose and porous.

### Characterization of zinc coating

**Figure 7:**
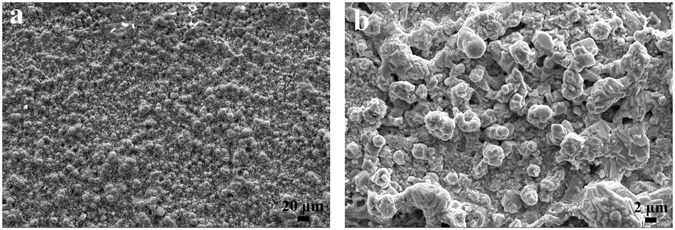
Surface morphology of zinc coating obtained by EPP under different voltages: (**a**) 150 V and (**b**) 180 V.

After EPP pretreatment of the steel at an applied voltage of 120 V for 60 s, the steel is further modified in 15 wt% ZnSO_4_ solution by means of EPP. Figure 7 represents the surface images of zinc coating formed on the steel surface with EPP applied voltages of 150 and 180 V for 60 s. When the surface coating of EPP treatment at an applied voltage of 150 V for 60 s, there is a loose zinc layer with the co-existence of needle-like dendrites and small pellets on the steel surface. Composition analysis of the surface coating (Table 1) reveals that the content of zinc was 61.52 at% besides quantities of Fe and O, indicating that the zinc layer could not completely cover the surface. These results verify that the EPP treatment can form zinc coating on the steel surface in a highly efficient way. During the coating process, a hydrogen evolution reaction likewise occurs under the effect of a breakdown voltage, creating the generation of hydrogen plasma in a thin electrolyte layer just above the surface of 304 stainless steel. Most of zinc cations presenting in the electrolyte can be attached to the hydrogen bubbles and migrated toward the surface of 304 stainless steel. Thus, zinc coating is continuously formed at a high rate over the entire sample surface via adsorption transport and subsequent collapse of hydrogen bubble.

**Table 1:** Surface composition of zinc coatings shown in Fig. 7(a) and (b).

	Zn (at. %)	Fe (at. %)	O (at. %)
a	61.52	2.41	36.07
b	99.71	0.29	0.00

In the case of high applied voltage of EPP treatment, i.e. 180 V, it can be observed from Fig. 7(b) that a compact layer of zinc coating with a relatively uniform distribution grows on the sample surface. In the meantime, zinc content reaches 99.71 at%, suggesting the complete coverage and homogeneous distribution of zinc element on the entire surface. This can be explained that high applied voltage provides considerable hydrogen plasma and forceful energy to rapidly migrate the attached zinc cations. Hence, zinc coating with a full coverage can be effectively obtained on the 304 stainless steel by means of EPP for only 60 s.

**Figure 8:**
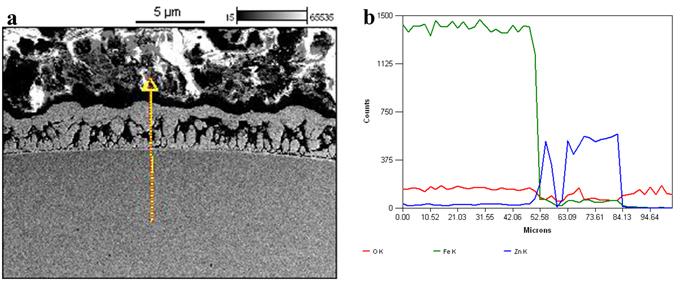
(**a**) Cross section morphology and (**b**) line scanning spectrum of zinc coating on 304 stainless steel.

Figure 8 exhibits the SEM cross-sections of the zinc coatings formed at an applied voltage of 180 V for 60 s, along with EDS line scans across the surface coatings. It can be seen that the zinc coating has a relatively uniform thickness of about 0.5 μm under the effect of EPP plasma. These results further demonstrate that EPP modification can successfully deposit zinc coating on 304 stainless steel in a highly efficient and effective way.

## Experimental

### Materials

304 stainless steel was supplied by Baosteel Group Corp with chemical composition listed in Table 2. Samples of the stainless steel were cut into pieces of 30 × 20 × 1 mm in size. Afterwards, all specimens were ground with progressively finer SiC paper (180, 400, 800, 1200, 2400, and 4000 grit) to minimize the effect of incidence angle. They were thoroughly washed and ultrasonically cleaned in distilled water and absolute ethanol for 10 min, and then dried in air. Further, sodium bicarbonate and zinc sulfate were purchased from J & K Chemical Technology.

**Table 2:** Chemical composition of 304 stainless steel (wt%).

C	Si	Mn	P	S	Cr	Ni
≤0.07	**≤1.0**	**≤2.0**	**≤0.035**	**≤0.03**	**17.0~19.0**	**8.0~11.0**

### EPP treatment

The electrode device and power source we used in the experiment is depicted in Fig. 2. The electrolyzer consisted of a silica glass container with the size of 20 × 10 × 15 cm. An anode supply was connected to two parallel graphite plates, and the sample of 304 stainless steel was used as a cathode (see Fig. 1). 7.5 wt% sodium bicarbonate and 15 wt% zinc sulfate solutions prepared with ultra-pure MilliQ water (resistance >18 MΩ cm^−1^) were used as electrolytes for the pretreatment of the steel surface and the formation of zinc coating, respectively.

In the case of surface pretreatment of the steel, the input voltage was set at 110, 120, and 130 V, and the processing time was varied in the range of 0–180 s for optimization of surface profile. The temperature of NaHCO_3_ electrolyte with an initial value of 70 °C was monitored by using a k-type thermocouple along with time. All samples were respectively washed with water and ethanol in ultrasound for three times, and dried in air prior to characterization analysis and further coating. For the formation of zinc coating, the obtained samples above were soaked in 15 wt% ZnSO_4_ solution for 60 s. Afterwards, EPP was operated by controlling an applied potential to 150 and 180 V, respectively, for 60 s.

### Characterization

The morphological features of 304 stainless steel and zinc coating were observed with a ZEISS SUPRA55-FESEM instrument, while composition analyses of the samples’ surfaces were performed via energy dispersion spectrometry (EDS). XRD patterns were examined on a Rigaku D/max 2500 instrument equipped with Cu Kα radiation. The three-dimensional (3D) surface morphology of the samples was characterized by white-light interferometer (State Key Laboratory of Tribology, Tsinghua University) with minimum vertical resolution of 0.8 nm. We used the optical interference method for quantitative measurement and our in-house system for the 3D surface texture images as well as for measuring the microscopic linear height measurement and average surface roughness (Ra). We used a Nano Indenter II to test the hardness of 304 stainless steel after EPP. The final hardness of 304 stainless steel was determined through the average of seven different positions along the radius from the surface to the core on a cross-section of each steel after surface pretreatment.

## Electronic supplementary material

Supplementary information
