# Supplementary material for: Electrolytic plasma processing-an innovative treatment for surface modification of 304 stainless steel
Source: Sci Rep. 2017 Mar 22;7:308. doi: 10.1038/s41598-017-00204-w (PMC5428051; doi:10.1038/s41598-017-00204-w)
Supplement: Supplementary file 1 — Supplementary information [file 41598_2017_204_MOESM1_ESM.pdf]

Electrolytic plasma processing-an innovative treatment for surface modification of 304 stainless steel

Wanyuan Gui[a], Junpin Lin[a]*, Guojian Hao[a], Yuhai Qu[a], Yongfeng Liang[a], Hui Zhang[b]*

Surface modification of 304 stainless steel may serve as theoretical guidance for the further development and application of EPP. There is an obvious enhancement in surface roughness of 304 stainless steel after EPP pretreatment. A layer of compact zinc coating with a thickness of approximately 0.5 μm can be obtained on the 304 stainless steel by means of EPP for only 60 s. Covering by homogeneous gas film and the breakdown voltage are two important parameters of formation of liquid phase plasma. Solution volatilization caused by joule heat and solution hydrodynamic instability caused by solvent electrolysis are two important mechanism of plasma formation.

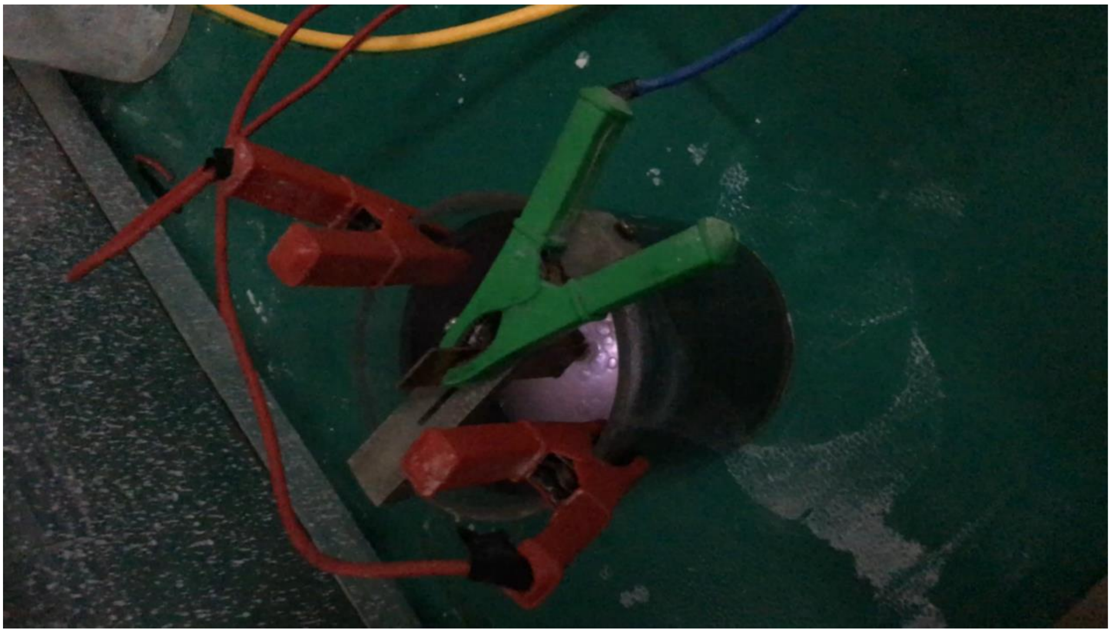

Figure. S1 The process of surface modification of 304 stainless steel by electrolytic plasma processing.
